# E. Pasteur’s Experiments on Charbon

**Published:** 1882-03

**Authors:** 


### E. Pasteur's Experiments on Charbon.

M. Pasteur has now an apportunity of practically testing the truth of his theory as to the protection of animals from charbon by their inoculation with the artificially cultivated virus of that disease. On the 5th ultimo, the farm of a veterinary surgeon at Pouilly-le-Fort, sixty sheep were placed at M. Pasteur's disposal. Ten of these sheep were left untouched, in order that they might later on serve for comparison. Of the remaining fifty, twenty-five were marked with a hole in their ears, and were inoculated, the first time on the 5th of May, and the second on the 17th. On May 31, none of the inoculated sheep had lost fat, or spirits, or appetite. On May 31, the fifty sheep were taken, without distinction, and inoculated with the strongest virus. M. Pasteur predicted that, on the 2d instant, the twenty-five sheep not inoculated would be dead, and that the inoculated animals would show no symptoms of sickness; and accordingly, on that day, a number of spectators, including the President of the Agricultural Society of Melun, the Prefect of the Department, and the Director of Agricultural Matters at the Ministry of Agriculture and Commerce, assembled to witness the result. The prophecy of M. Pasteur was exactly fulfilled. At two o'clock twenty-three of the sheep which had not been inoculated were dead. At three o'clock the twenty-fourth died, and the twenty-fifth an hour later. The twenty-five inoculated animals were quite sound, and in perfect health. Only one of them was feverish; and the fever, which was caused by the animal having designedly been inoculated with too strong a dose of the virus, speedily disappeared. The twenty-five carcases have been buried in a fixed spot; and, on the infected grass which will grow over it, experiments are to be made with inoculated and non-inoculated sheep. M. Pasteur's discoveries in this direction are being amply corroborated by English experience, and, if the matter be properly taken up, the agricultural interest will have one less difficulty to contend against, since it ought to be practicable to protect herds against charbon much in the same way that human beings can protect themselves against small-pox by vaccination.—*Chicago Med. Journal and Examiner.*

